# Diagnosis and dilemma: Clinician experiences of the use of ‘borderline personality disorder’ diagnosis in children and adolescents

**DOI:** 10.1002/pmh.1541

**Published:** 2022-04-08

**Authors:** Rose Papadopoullos, Paul Fisher, Adrian Leddy, Sarah Maxwell, Jo Hodgekins

**Affiliations:** ^1^ Department of Clinical Psychology, Norwich Medical School University of East Anglia Norwich UK; ^2^ The Family Psychologist Kidderminster UK; ^3^ Cranbrook Centre Northgate Hospital Great Yarmouth UK

## Abstract

Borderline personality disorder (BPD) diagnosis in adolescents is a relatively recent concept and a fast‐emerging research area. Regarded by some as controversial, it is important for research to provide greater understanding of differing perspectives and their impact on the use of this diagnosis. Perspectives of 13 clinicians (therapists, psychiatrists and mental health nurses) were explored, to provide a contemporary understanding of perceptions and use of BPD diagnosis within child and adolescent mental health services in England. A particular focus was to explore dilemmas faced by clinicians and how these dilemmas were negotiated. This research took a qualitative, social constructionist approach to explore the in‐depth views and experiences of each participant. Interviews were analysed using thematic analysis, to seek out patterns and commonalities across these clinical perspectives. Three overarching themes were generated: ‘Who holds the power?’, ‘Dilemmas within the multidisciplinary team (MDT)’ and ‘The weightiness of making this decision’. Professional opinions of an adolescent BPD diagnosis are influenced by dominant and less dominant mental health discourses, including the impact of power, and availability of resources within the service context. The role of meaningful collaboration with young people, clinical implications and directions for future research are discussed.

## INTRODUCTION

The UK's mental health system, and many others globally, is predominantly situated within a ‘medical‐model’ understanding of distress, drawing from diagnostic manuals to describe and categorise people's difficulties (Cooke et al., 2019). From this perspective, the diagnosis ‘borderline personality disorder’ (BPD) is characterised by intense and changeable emotions, no stable sense of identity, and long‐term difficulties forming and maintaining healthy relationships (American Psychiatric Association, 2013). Since BPD was first described (American Psychiatric Association Committee of Nomenclature and Statistics, 1980), it has been a diagnosis primarily considered within adult populations. Over the last decade or two, there has been an increase in research into ‘adolescent’ or ‘emerging’ BPD, and the diagnostic manual *International Classification of Diseases 11th Revision* (World Health Organization, 2018) describes diagnosis in under 18s for the first time (bringing it in line with BPD criteria in the *Diagnostic and Statistical Manual of Mental Disorders, 5th ed*.). Indeed, the current dominant body of research argues for the validity of an adolescent BPD diagnosis (Miller et al., 2008; Winsper et al., 2016), and there is evidence that early diagnosis provides a pathway towards earlier intervention (Chanen et al., 2008), with a range of therapeutic interventions being developed globally. However, the most recent systematic review of the effectiveness of these adolescent early interventions for BPD, published in 2021, has reported that current research is generally of poor quality, with interventions showing limited benefits over and above control interventions, and high rates of participants dropping out (Jørgensen et al., 2021). In studies from 2011 and 2013, a majority (64%) of 52 UK psychiatrists believe that adolescent BPD diagnosis is inappropriate, invalid or harmful (Griffiths, 2011), and less than 10% of 566 psychologists in the Netherlands and Belgium said they would use BPD diagnosis for adolescents, for reasons including stigma, and symptoms during adolescence being unpredictable (Laurenssen et al., 2013). These concerns may reflect older and more stigmatised attitudes to the present day; however, research has demonstrated that BPD symptom variation fluctuates widely throughout adolescence (Conway et al., 2017) and BPD diagnosis has been widely stigmatised (Aviram et al., 2006), even within more recent research from 2017 showing that health professionals still show reduced empathy towards adults with this diagnosis and negative expectations towards their recovery (Chartonas et al., 2017).

Although some adults describe a diagnosis of BPD as a normalising and validating experience, and a ‘turning point’ in their recovery (partly through access to evidence‐based treatments) (Ng et al., 2019), others have described discrimination, feelings of self‐judgement, rejection, hopelessness and a sense of the diagnosis being all‐encompassing (Horn et al., 2007; Ng et al., 2019). In part, these varied experiences are constructed through the attitudes and language used in discussions between mental health professionals and users of a service (Femdal & Knutsen, 2017; Lester et al., 2020; Sewell, 2018), and can become most negative in settings where staff feel underskilled or under‐resourced (Albaek et al., 2018; Lester et al., 2020). One way of challenging negative perceptions of mental health is to view distress through a psychosocial framework (British Psychological Society, 2011), to understand all behaviour and experience as meaningful responses to adverse events (Johnstone et al., 2018). However, ‘psychosocially minded’ psychologists can find it difficult to work this way within the UK's medical‐model NHS (Cooke et al., 2019), and the avoidance of diagnostic labels all together can unintentionally discourage an open discussion of the difficulties someone is facing (Weiste et al., 2021).

Many of these studies were conducted before the publication of the ICD‐11 and therefore before the increased shift in how a BPD diagnosis may be used with children and adolescents. This project addresses the need to update our understanding of how clinicians perceive and use BPD diagnosis within child and adolescent mental health services, by exploring two main areas:

1. Based upon their experiences to date, how valid and useful do clinicians believe a diagnosis of BPD is for young people under 18 years old?
2. Are there any dilemmas faced by clinicians regarding use of this diagnosis, and how are such dilemmas negotiated?

## MATERIALS AND METHODS

This research took a qualitative, social constructionist approach to explore in‐depth views and experiences of participants, acknowledging the role of society, culture and social interactions on people's behaviour and how we understand the world (Gergen, 1985). A central position in social constructionism is that people co‐create meaning within the context of these social influences (Berger & Luckmann, 1991). In research, the subjective interrelationship between the researcher and interviewee plays an important role in the co‐creation of this meaning and knowledge (Mills et al., 2006). Findings provide a unique insight into the experiences of these participants at this point in time, rather than striving for generalisable or replicable data (Burr, 1995).

### Ethical approval

Ethical approval was provided by the University of East Anglia Research Ethics Committee (Reference 201718‐24). As recruitment was within England's National Health Service (NHS), approval from the Health Research Authority in England was also sought and granted (IRAS ID 212121).

### Participants

Thirteen clinicians participated in interviews, including five psychiatrists, four therapists (including CBT therapists, family therapists and psychologists) and four mental health nurses. This included six male participants and seven female participants. As many of these local teams are small in terms of staff numbers (in particular only one or two psychiatrists within each team), it was felt that participants might be identifiable if further demographic information was reported.

Recruitment to the study was advertised verbally to clinicians working in three community and one inpatient child and adolescent mental health services (CAMHS) within one NHS mental health trust in England during multidisciplinary team (MDT) meetings. Interested staff members read an information sheet before giving informed consent to participate in the research and for their data to be used. Flexible times, dates and locations for interviews were offered.

Sample size was intended to be large enough to provide new understandings, while being small enough to allow for a deep individual focus (Sandelowski, 1995). Research has shown that open interview questions tend to produce richer and more useable qualitative data (Ogden & Cornwell, 2010), meaning fewer participants are needed to reach a deep understanding of the topic. In addition, with a relatively homogenous sample and focussed research aims, it has been argued that 12 interviews can provide sufficient data (Guest et al., 2006). Finally, we considered the number of participants within other qualitative research in this area (e.g., nine out of 12 papers reported in a recent qualitative systematic review had 10 or less participants each [Lester et al., 2020]) and what was a realistic recruitment aim in terms of the number of clinicians locally. Overall, 13 participants were deemed appropriate.

### Interviews

A semi‐structured interview schedule aimed to open up novel areas of discussion, especially around the experience of dilemmas. Local clinicians were consulted regarding topic feasibility, acceptability and clarity of the wording used. The final schedule was not intended as a checklist of questions, but as a flexible open‐ended tool to help guide the researcher and participant through the interview topics. The priority was to ensure the participant was able to talk in‐depth about experiences that felt relevant to them. Prompts such as ‘can you tell me more about that?’ or ‘do you have an example of that?’ were used throughout to gain a deeper level of description and understanding. Interviews were conducted face to face, by a trainee clinical psychologist, lasting for an average of 1 h. Interviews were audio recorded and transcribed orthographically to provide a verbatim representation of the conversation (Braun & Clarke, 2006).

### Analysis

Thematic analysis (TA) allows for an in‐depth analysis moving beyond one individual's experience to seek out patterns and commonalities across the broader data set (Braun & Clarke, 2006). TA followed Braun and Clarke's six stages (Braun & Clarke, 2006) taking an inductive ‘bottom‐up’ approach, building on observations from the raw data without a theoretical model guiding the analysis process. First, listening to audio while reading each transcript ensured familiarisation and closeness to the data. Next, taking each transcript line by line, all meaningful data extracts were coded by hand, continually referring to surrounding text to prevent data being coded out of context. In the naming of codes, effort was made to remain grounded in the participants' own words. A research supervisor (JH) also coded several interviews, allowing for useful reflections around similarities and differences in the coding process. Once all transcripts had been coded at this level, the process of grouping codes began. As TA is an iterative process (Braun & Clarke, 2006), the combining of codes was not fixed. Each grouping or theme was revisited in the context of how others were being formed, aiming to organise the codes, sub‐themes and themes until each theme felt cohesive and distinct from other themes. This meant frequently moving up and down the ‘levels’ of themes–sub‐themes–codes and referring back to the interview transcripts to ensure that groupings made sense and that the meanings being generated remained grounded in the participants' accounts. Striving to achieve ‘data saturation’ is not consistent with the reflexive TA methodology (Braun & Clarke, 2021); however, it was felt that the data collected were suitably rich to allow for a robust and in‐depth analysis. NVivo software was used as an aid to analysis (QSR International Pty Ltd, 2015), though pen and paper were also used, particularly during the later stages of refining themes and sub‐theme.

From a social constructionist approach, researchers reflect on their own positions (experiences, assumptions and investment in the project) to foster transparency and openness to preconceptions (Elliot et al., 1991). A reflective research diary and ‘memos’ were kept throughout the study, to notice and reflect on the process of data collection and analysis. Supervision by qualified clinical psychologists provided further opportunity to reflect on preconceptions (O'Brien et al., 2014) and also added further power dynamics to be mindful of. Ultimately, supervision and the research diary supported a richer and more thoughtful analysis (Sullivan et al., 2012) as well as ensuring dependability and transparency of the qualitative analysis process (Sandelowski, 1986).

## RESULTS

Three core themes, each made up of two to three sub‐themes, were formed during analysis, as outlined in Table 1.

**TABLE 1:** An overview of themes and sub‐themes

Theme	Sub‐themes
Who holds the power?	Empowering clinicians Loss of autonomy Collaborating—or not—with the young person
Dilemmas within the MDT	Feels like a bit of a battle Navigating through team conflict
The weightiness of making this decision	It's a particularly difficult decision in adolescence ‘We're all coming from the same page’ Impact on patient care Detrimental impacts on the young person

Abbreviation: MDT, multidisciplinary team.

### Who holds the power?

There was a sense of empowerment for clinicians (particularly psychiatrists) being able to have a positive impact on a young person's prognosis, though this comes alongside perceived service shortcomings. In addition, participants' experiences highlight a juxtaposition relating to this diagnosis; whereas clinicians become more empowered, young people may become less so.

#### Empowering clinicians

On one hand, participants spoke about how new clinical research has informed how mental health professionals understand and treat BPD. Feeling empowered in this way means use of BPD diagnosis during adolescence becomes perceived to be less daunting and more useful:

I think generally people's perceptions of BPD is changing a bit … the sense that you can treat it, so you can advocate that way. There are treatments and there's an evidence base for them so, it feels less hopeless maybe than it did before. (Psychiatrist)

Alongside this, there was a feeling that empowerment is dependent on how stretched resources are. For example, there was a frustration with perceived cuts in funding for NHS mental health services (‘we know what works but, um, the funding isn't there’ [psychiatrist]), and perceptions of BPD were therefore constructed within the context of what resources and experience were available within the team:

Where maybe there's a bit more of a culture of how to work with these cases maybe [clinicians] feel a bit more empowered to work with them and there's interventions that are available that they can offer that are helpful … professionals in those teams are a bit more positive about [giving young people a BPD diagnosis]. (Psychiatrist)

#### ‘It's not me, it's my disorder’

Although some clinicians may feel more empowered, BPD diagnosis was seen to disempower the young people who are given this diagnosis:

You kind of externalise [their difficulties] into a diagnosis and [they] say … ‘I don't have control, I've got this diagnosis’. (Therapist)

Similarly, a nurse described hearing statements like ‘‘Oh, it's not me, it's my personality disorder’. There was also a sense that this diagnosis can lead people into a ‘fantasy’ (therapist) of a ‘magical cure’ (psychiatrist), and young people or their family members were quoted as saying ‘It's too hard. I want a quick fix … I want you to rescue me’ (nurse). Participants emphasised how they try to both empathise and motivate young people, attempting to counterbalance this loss of autonomy: ‘this isn't your fault, but it is your responsibility’ (nurse).

#### Collaborating—or not—with the young person

Taking a collaborative approach was seen as important in making sure not to lose the young person behind their diagnosis: ‘they're not all emotional unstable personalities. They're Becky or John or Peter’ (nurse). Psychiatrists—the only professional group in this study who formally gives diagnoses—spoke about young people playing an important role in ‘helping me figure out if that's the right thing’. However, where a crisis has occurred or risks are high, psychiatrists felt their decision would ‘trump the patient’, emphasising how easy it can be for clinicians to occupy an expert position that negates true collaboration. This power dynamic was also seen in subtle ways, such as one psychiatrist's assumption that a young person would be able to challenge the use of this diagnosis if they did not agree with it:

Psychiatrist It would be interesting to see if I get [some]one where they say ‘no no, you're off‐piste completely’ [laughs] I guess if they say that then I'll say ‘fine … it must be something else’ and try and think of it in another way.

Interviewer But that's not happened?

Psychiatrist Not yet, no. No.

### Dilemmas within the MDT

Participants described a polarised ‘medical vs. psychological’ approach to BPD diagnosis, making it a difficult topic to talk about. This was experienced by participants as part of the dynamics within their MDTs and was also evident within the research interviews themselves.

#### Feels like a bit of a battle

There was a perception that in mental health services, ‘the bias is still on a medical model’ (psychiatrist), and therapists and nurses describe a ‘powerful tension’ (therapist) within the team; a ‘push and pull’ (therapist) between the medical and the psychological, which as this nurse described, can become confusing for some:

You're trying to make the right decisions and we will have input from the psychology department … and then I guess we are pulled in the other direction when we are attending medical reviews … you'll kinda of get wrapped up with ‘is it okay?’, ‘am I even more confused than when I started?’ [laughs]. (Nurse)

Participants described this as pervasive, with frequent ‘debate and consternation’ (psychiatrist). This dynamic also arose during some interviews, for example, where participants seemed to anticipate conflict and attempt to stop or redirect particular questions:

Psychiatrist Should ask society this question rather than me. But I'm not blind to stigma. I'm just recognizing that it is so bad.

Interviewer Mm, mm, and so you think its society, that the stigma from‐

Psychiatrist Well, I'm from society and so are you. You can ask yourself.

#### Navigating through team conflict

Participants mentioned strategies they use to make sense of, and work within, discomfort within the team. One strategy was to frame conflict as helpful: a ‘sign of a healthy team’ (psychiatrist). Participants also spoke about finding allies and supporters, seeking ‘respect and validation from colleagues … “you are doing the right kind of things” ’ (therapist)’. However, there was also a sense of hesitance, being ‘very careful about how we talk about things’ (nurse). A therapist says ‘I won't go and campaign against [BPD diagnosis] … I just keep my head down and do what I'm supposed to do’. This hesitance was also noticed during interviews, with participants and the interviewer sometimes struggling to find the ‘right’ words.

It is possible that this avoidance of conflict prevents nuanced, open and honest discussions at times. For example, one nurse said ‘we're all saying the same thing’ in disagreeing with the use of BPD diagnosis in adolescents; however, other interviews with people in the same team showed that this is not the reality. A psychiatrist who initially said their team were all ‘on the same page’ struggled to explain this further:

Psychiatrist When we do talk about it, we are on the same page about it, um‐ [pause].

Interviewer In what sense?

Psychiatrist That I do not‐ Well, the colleagues that I've spoken to in this, uh, this um, we all—[pause]. Let me talk about borderline personality, we all know what it is.

### The weightiness of making this decision

Clinicians spoke about difficult dilemmas around whether to diagnose a young person with BPD or not. They spoke of a push and pull between avoiding the use of a potentially harmful ‘label’ while acknowledging helpful aspects of BPD diagnosis.

#### It is a particularly difficult decision for adolescents

Clinicians were clear about being cautious with this diagnosis in under 18s: ‘the stakes are high … you have to be careful about giving that diagnosis’ (therapist), especially as ‘brains are still developing’ (nurse). Some described themselves as ‘less comfortable’ (psychiatrist) with BPD diagnosis at this age, whereas others passionately questioned its meaning: ‘what does that even mean? A personality disorder before the age of 18. What are you even saying?’ (nurse). A psychiatrist, who had already discussed some positives to the use of this diagnosis, stated firmly:

But it's definitely not good for somebody that's going through … adolescent change and dysregulation … to be given a diagnosis would be damaging and harmful and should not happen.

However, this had to be weighed up alongside other considerations such as the view that ‘withholding that diagnosis is potentially harmful’ (psychiatrist). For example, when clinicians ‘beat around the bushes [to avoid giving a BPD diagnosis]’ (psychiatrist), the young person may instead attract multiple inappropriate diagnoses. In addition, some felt that the diagnosis was a way to help the young person understand themselves. A psychiatrist recalls a young person saying ‘gosh for the first time I've read something that described how I feel’.

It seemed that the weightiness of this decision can lead to a reluctance to make this decision alone:

Times have been [when the team has] said ‘why don't you just give them the diagnosis already! You're waiting too long’ [laughs] … So ok you do think I'm being too cautious? They go ‘yes!’ You know, ‘go for it!’. (Psychiatrist)

In response to these difficulties, the use of ‘criteria’ was seen to help clinicians back up their decision. For some, meeting the criteria helped them justify a diagnosis; for example, one psychiatrist said ‘It's useful if they satisfy the criteria’, and another said ‘they really meet criteria [laughs] it's obvious that's what they had’.

#### Impact on the young person and their care

Many perceived a diagnosis of BPD to be ‘just the tip of the iceberg’ (nurse); the perception being that this diagnosis is often not very useful clinically and by ‘label[ing] the adolescent as the problem’ (therapist) you miss opportunities to intervene more systemically. Participants also raised concerns about the permanency of the diagnosis: ‘I would imagine that for a lot of people it stays with them forever … I think it's very rare that those sorts of labels become removed’ (therapist), and feelings of shame or blame that young people internalise: ‘I‐I am broken. See, I have this label’ (nurse). However, the NHS was described as a diagnostic system, with diagnoses helping professionals to communicate and seen as essential in advocating for the young person to ‘pull services in’ (psychiatrist). Some participants felt uncomfortable within this position, with a nurse reluctantly saying ‘for social services, for funding panel, they need a diagnosis. They can't get funding through if they haven't got a diagnosis’.

## DISCUSSION

This research explored clinician perspectives of the diagnosis of adolescent BPD. Three themes emerged from the data: ‘Who holds the power?’, ‘Dilemmas in the MDT’ and ‘The weightiness of this decision’. Running through these results were how the perspectives around BPD diagnosis may be socially constructed through the interactions between dominant and less dominant mental health discourses, power dynamics within MDTs and the personal experiences and views of professionals.

BPD diagnosis was a difficult topic to talk about, and use of language seemed to be cautiously negotiated by participants. Within the interviews themselves, there were times when participants found it hard to choose the right words, spoke tentatively about their opinion, contradicted themselves or wanted to know the researcher's opinion (perhaps as a way of exploring what they ‘could’ or ‘could not’ say). The interviewer felt this too, noticing her own struggle to find words that felt ‘right’ at times where the interpersonal dynamics within the interviews created a sense of unease, or lack of safety to explore certain topics. This dynamic closely mirrored the hesitance clinicians spoke of in causing conflict within their team, with several preferring to go along with the dominant medical‐model systems rather than finding ways to express alternative views. Indeed, language within a medical‐model discourse can establish inherent power imbalances, not just between clinicians and users of services, but also between professional groups (Sewell, 2018).

This discomfort is reiterated by Cooke et al. (2019) emphasising how these challenges are not unique to these participants, especially where views fall outside of the dominant medical model. For the few who spoke confidently about their viewpoint, it was perhaps easier for them to share these views with someone outside of their team, or who, as a trainee may have been perceived to fall hierarchically below them. Shifting power dynamics noticed during the interviews may also reflect how debates between professionals play out within teams. Clinicians often move into the expert position when they feel a need to take back power or control (Femdal & Knutsen, 2017). It is possible that this strategy, alongside others' desire to avoid conflict, may result in a loss of nuanced and open discussion and that false sense of everyone ‘being on the same page’.

Despite this, most participants spoke of wanting to understand young peoples' experiences in meaningful ways, though research suggests that clinicians are not always resourced enough to do this (Braun & Clarke, 2006). Indeed, participants reflected on the culture and resources within their teams and described a sense of powerlessness working in services they perceived to be under‐resourced. Situated within the social and political context of England's NHS, there have been recent commitments to early intervention in children and young people's mental health (NHS England, 2019), and specialist intervention for BPD is recommended for adults and adolescents (National Institute for Health and Care Excellence, 2009; Snowden & Kane, 2003). However, as participants noted, resources and service provision are inequitable across the country (Dale et al., 2017), raising an important question about the value of BPD diagnosis in services where clinicians are not able to/do not feel empowered to provide appropriate support. Clinicians working with more resources or within specialist services might bring a different attitude to this diagnosis (Lester et al., 2020).

Participants also raised concerns about negative impacts BPD diagnosis could have on a young person, including losing hope for the future and losing any sense of identity outside of ‘BPD’—views that have been reiterated through the lived experiences of some adults with a diagnosis of BPD (Horn et al., 2007; Ng et al., 2019). The perceived loss of autonomy, which a BPD diagnosis can bring to a young person and to those around them, was emphasised by all participants. In response to this, participants felt they had a responsibility to promote engagement and autonomy in adolescents, which, ironically has been shown to maintain an imbalance of power by situating the clinician within an empowered role of ‘motivator’ or creator of change and inviting service users into either a subordinate ‘dependent’ position or a disengaged ‘resistant’ position (Femdal & Knutsen, 2017).

Several participants expressed the same concern as psychologists in Laurenssen et al.'s (2013) work; that diagnosis was inappropriate given adolescents' developmental context and the instability of symptoms at this age (Conway et al., 2017). However, they also feared that reluctance to diagnose BPD could simply mean that adolescents end up inappropriately diagnosed with multiple other diagnoses, thereby preventing a meaningful understanding of their difficulties and access to appropriate treatment. Research suggests that there could be a ‘subgroup’ of adolescents whose BPD symptoms do remain stable into adulthood (Miller et al., 2008; Winsper et al., 2016). The pressure felt by clinicians to be able to discriminate between these adolescents was clearly huge, and several described strategies to avoid sole responsibility for decisions, including referring back to ‘criteria’ or deferring to the team. Underlying this for some participants was optimism that early diagnosis of BPD could enable access to effective early intervention. Indeed, some adults have described diagnosis as an important ‘turning point’ in their recovery journey (Ng et al., 2019), partly through access to these evidence‐based treatments. However the evidence base for adolescent BPD early intervention is still lacking (Jørgensen et al., 2021), and some participants felt uncomfortable with the perceived need for what was described as a diagnostic ‘label’ if a shared understanding of the young person can be reached through formulation instead (British Psychological Society, 2011; Johnstone et al., 2018). This was also described by clinicians in Cooke et al.'s (2019) study who negotiated dilemmas by collaborating with young people themselves. However, this collaboration was often tied up within power dynamics meaning that despite clinicians' best intentions to collaborate, this is not always achieved in a meaningful way.

### Limitations and future directions for research and clinical practice

By taking a social constructionist stance, the knowledge created through this research drew upon the context within which interviews took place and the unique interactions that occurred between interviewer and participants. Overall, we have explored, in‐depth, a phenomenon not previously described in the research: the way dilemmas arise and are negotiated by clinicians working with adolescents who could be given a BPD diagnosis. Contextual factors, such as team culture, skills and perceived resources, had an impact on the way clinicians interpret this diagnosis, on the language used throughout these interviews and on the final interpretations as presented in this paper. Clinicians within other contexts (especially well‐resourced or specialist settings) are likely to have alternative views and perspectives, which would be interesting to explore in future research. There was an emphasis throughout analysis to ensure closeness to the data and credibility in the findings presented. Nevertheless, there will of course be unnamed ideas that may have felt unsafe or unacceptable for participants to express during their interview, something that is also reflective of how clinicians felt within their teams. Interestingly, although a range of professions participated, there was an over‐representation of psychiatrists in comparison with the proportion of other professional groups working within these services. This may have reinforced the sense of there being a hierarchy within these services, and the power of psychiatry and the medical model as a dominant professional voice within mental health research (Cooke et al., 2019; Haslam & Lusher, 2011). Future research would benefit from a focus on exploring the perspectives of lesser heard voices; most critically, the views and experiences of young people who could receive a BPD diagnosis, and their families, are missing from this research. The variety of both positive and negative experiences in adults (Horn et al., 2007; Lester et al., 2020; Ng et al., 2019) reinforces how essential it is that these perspectives need to be understood in their own right, for example, by exploring young people's perceptions of the words ‘borderline personality disorder’, and the role this diagnosis plays in understanding their sense of self and their recovery journey.

Finally, this research emphasises a need to build awareness amongst clinicians of the impact that their power and professional role have on their ability to meaningfully collaborate about BPD diagnosis with young people. Previous studies have shown that ongoing interaction between professionals and service users, and meaningful involvement from young people in decision making (e.g., through third‐sector organisations such as Emergence, 2019), and collaborative ways of designing services (Larkin et al., 2015) might be ways in which some of the power imbalance can be reduced.

## CONFLICT OF INTERESTS

No potential competing interest was reported by the authors.

## FUNDING INFORMATION

Research was completed as part of a doctoral thesis on the Doctoral Programme in Clinical Psychology at Norwich Medical School, University of East Anglia.

## ETHICS STATEMENT

Ethical approval was provided by the University of East Anglia Research Ethics Committee (Reference 201718‐24). As recruitment was within England's National Health Service (NHS), approval from the Health Research Authority in England was also sought and granted (IRAS ID 212121).

## Data Availability

The data that support the findings of this study are available from the corresponding author upon reasonable request.
